# Similarity Measure and Entropy of Fuzzy Soft Sets

**DOI:** 10.1155/2014/161607

**Published:** 2014-06-15

**Authors:** Zhicai Liu, Keyun Qin, Zheng Pei

**Affiliations:** ^1^College of Mathematics, Southwest Jiaotong University, Chengdu, Sichuan 610031, China; ^2^Center for Radio Administration and Technology Development, Xihua University, Chengdu, Sichuan 610039, China

## Abstract

Soft set theory, proposed by Molodtsov, has been regarded as an effective mathematical tool to deal with uncertainties. Recently, uncertainty measures of soft sets and fuzzy soft sets have gained attentions from researchers. This paper is devoted to the study of uncertainty measures of fuzzy soft sets. The axioms for similarity measure and entropy are proposed. A new category of similarity measures and entropies is presented based on fuzzy equivalence. Our approach is general in the sense that by using different fuzzy equivalences one gets different similarity measures and entropies. The relationships among these measures and the other proposals in the literatures are analyzed.

## 1. Introduction

To solve complicated problems in economics, engineering, environmental science, and social science, methods in classical mathematics are not always successful because of various types of uncertainties presented in these problems. In 1999, Molodtsov [1] introduced the concept of soft sets, which can be seen as a new mathematical tool for dealing with uncertainties. Concretely, a soft set is a parameterized family of subsets of the universe. The absence of any restrictions on the parameter in soft sets makes this theory very convenient and easily applicable in practice. Applications of soft sets in areas ranging from decision making problems to texture classification have surged in recent years [2–5].

Works on soft set theory are progressing rapidly. Maji et al. [6] defined several algebraic operations on soft sets and made a theoretical study on the theory of soft sets. Based on [6], Ali et al. [7] introduced some new operations on soft sets and improved the notion of complement of soft set. They proved that certain De Morgan's laws with respect to these new operations hold in soft set theory. Qin and Hong [8] introduced the notion of soft equality and established lattice structures and soft quotient algebras of soft sets. Maji et al. [9] initiated the study on hybrid structures involving soft sets and fuzzy sets. They proposed the notion of fuzzy soft set as a fuzzy generalization of classical soft sets and some basic properties were discussed. Afterwards, many researchers have worked on this concept. Various kinds of extended fuzzy soft sets such as generalized fuzzy soft sets [10], intuitionistic fuzzy soft sets [11, 12], interval-valued fuzzy soft sets [13], vague soft sets [14], interval-valued intuitionistic fuzzy soft sets [15], and soft interval set [16] were presented. The combination of soft set and rough set [17, 18] is another interesting topic [19–22]. Furthermore, soft set theory has been applied to several algebraic structures such as groups [23], semirings [24], rings [25], BCK/BCI-algebras [26, 27], and BL-algebras [28].

The measurement of uncertainty is an important topic for the theories dealing with uncertainty. The similarity measure, distance measure, entropy in fuzzy set theory, and the relationships among these measures have been extensively studied for their wide applications in image processing, clustering, pattern recognition, case-based reasoning, and many other fields [29–40]. Majumdar and Samanta [41, 42] initiated the study of uncertainty measures of soft sets and fuzzy soft sets. Some similarity measures between soft sets and fuzzy soft sets were presented. Kharal [43] introduced some set operations based on distance and similarity measures for soft sets. Moreover, the new similarity measures were applied to the problem of financial diagnosis of firms. Based on the distance measures between intuitionistic fuzzy sets, Jiang et al. [44] proposed some distance measures between intuitionistic fuzzy soft sets and constructed some entropies on intuitionistic fuzzy soft sets and interval-valued fuzzy soft sets. Wang and Qu [45] proposed a similarity measure, a distance measure, and an entropy for vague soft sets. The relationships among these measures were analyzed. These studies present a preliminary, but potentially interesting research direction. However, some basic problems still need further investigation. Firstly, a fuzzy soft set is a parameterized family of fuzzy sets on the universe. Different fuzzy soft sets may have different parameter sets. The similarity measures presented in [42] are actually partial measures in the sense that they take only the fuzzy soft sets with the whole parameter set into account, although these measures can be extended to fuzzy soft sets with the same parameter sets. The same problem appears in the distance measures and similarity measures presented in [44, 45]. Secondly, in order to cope with different practical problems, it is necessary to find more new similarity measures and entropies and give a deep comparison with the proposals mentioned before. By the way, Min [46] introduced the concept of similarity between soft sets. Here the similarity is defined by a mapping between parameter sets. It is an extension of the equality for soft sets but not the similarity measure between soft sets.

The aim of this paper is to propose a new category of similarity measures and entropies for fuzzy soft sets based on fuzzy equivalences. Our approach is general in the sense that by using different fuzzy equivalences one gets different similarity measures and entropies. The paper is organized as follows. In Section 2, we recall some notions and properties of soft sets, fuzzy soft sets, and similarity measures for fuzzy sets. In Section 3, the axioms for similarity measure between fuzzy soft sets are proposed. Based on fuzzy equivalences, some similarity measures between fuzzy soft sets are presented. Furthermore, a comparative study of the similarity measures presented in this paper and that mentioned in the literatures is given. In Section 4, based on similarity measures, we construct some entropies for fuzzy soft sets. The paper is completed with some concluding remarks.

## 2. Overview of Soft Sets and Fuzzy Soft Sets

In this section, we recall some fundamental notions of soft sets, fuzzy soft sets, and similarity measures for fuzzy sets. See especially [1, 6, 9, 37, 47] for further details and background.

Let *U* be the universe set and *E* the set of all possible parameters under consideration with respect to *U*. Usually, parameters are attributes, characteristics, or properties of objects in *U*. (*U*, *E*) will be called a soft space.

**Definition 1 (see [6]).**

A pair (*F*, *A*) is called a soft set over *U*, where *A*⊆*E* and *F* is a mapping given by *F* : *A* → *P*(*U*).

In other words, a soft set over *U* is a parameterized family of subsets of *U*. *A* is called the parameter set of the soft set (*F*, *A*). For *e* ∈ *A*, *F*(*e*) may be considered as the set of *e*-approximate elements of (*F*, *A*). For illustration, we consider the following example of soft set.

**Example 2 .**

Suppose that there are six houses in the universe *U* given by *U* = {*h*_1_, *h*_2_, *h*_3_, *h*_4_, *h*_5_, *h*_6_} and *E* = {*e*_1_, *e*_2_, *e*_3_, *e*_4_, *e*_5_} is the set of parameters. *e*_1_, *e*_2_, *e*_3_, *e*_4_, and *e*_5_ stand for the parameters “expensive,” “beautiful,” “wooden,” “cheap,” and “in the green surroundings,” respectively.

In this case, to define a soft set means to point out expensive houses, beautiful houses, and so on. The soft set (*F*, *E*) may describe the “attractiveness of the houses” which Mr. X is going to buy. Suppose that *F*(*e*_1_) = {*h*_2_, *h*_4_}, *F*(*e*_2_) = {*h*_1_, *h*_3_}, *F*(*e*_3_) = {*h*_3_, *h*_4_, *h*_5_}, *F*(*e*_4_) = {*h*_1_, *h*_3_, *h*_5_}, and *F*(*e*_5_) = {*h*_1_}. Then the soft set (*F*, *E*) is a parameterized family {*F*(*e*_*i*_); 1 ≤ *i* ≤ 5} of subsets of *U* and gives us a collection of approximate descriptions of an object. *F*(*e*_1_) = {*h*_2_, *h*_4_} means “houses *h*_2_ and *h*_4_” are “expensive.”

It has been proved that fuzzy set and rough set are all special cases of soft set [23]. The theory of fuzzy sets initiated by Zadeh [47] provides an appropriate framework for representing and processing vague concepts by allowing partial memberships. Let *U* be a nonempty set, called universe. A fuzzy set *μ* on *U* is defined by a membership function *μ* : *U* → [0,1]. For *x* ∈ *U*, the membership value *μ*(*x*) essentially specifies the degree to which *x* belongs to the fuzzy set *μ*. There are many different definitions for fuzzy set operations. With the min-max system proposed by Zadeh [47], fuzzy set intersection, union, and complement are defined component-wise as follows:

(1) $$\begin{array}{c} \left(\mu \cap \nu \right)\left(x\right)=\mu \left(x\right)\wedge \nu \left(x\right),\\ \left(\mu \cup \nu \right)\left(x\right)=\mu \left(x\right)\vee \nu \left(x\right),\\ \mu^{c}\left(x\right)=1-\mu \left(x\right), \end{array}$$

where *μ*, *ν* are fuzzy sets on *U* and *x* ∈ *U*. *μ* is called a fuzzy subset of *ν* if *μ*(*x*) ≤ *ν*(*x*) for any *x* ∈ *U*. Clearly, the notion of fuzzy subset is a generalization of the notion of subset in classical set theory.

Maji et al. [9] introduced the concept of fuzzy soft set by combining soft set and fuzzy set.

**Definition 3 (see [9]).**

Let (*U*, *E*) be a soft space. A pair (*F*, *A*) is called a fuzzy soft set over *U*, where *A*⊆*E* and *F* is a mapping given by *F* : *A* → *F*(*U*); *F*(*U*) is the set of all fuzzy subsets on *U*.

**Definition 4 (see [9]).**

Let (*F*, *A*) and (*G*, *B*) be two fuzzy soft sets over a common universe *U*. Then one has the following.

1. (*F*, *A*) is said to be a fuzzy soft subset of (*G*, *B*), denoted by (*F*, *A*)⊆(*G*, *B*), if *A*⊆*B* and ∀*e* ∈ *A*, *F*(*e*)⊆*G*(*e*).
2. (*F*, *A*) is equal to null fuzzy soft set, denoted by *∅*_*A*_, if *F*(*e*) = *∅* for any *e* ∈ *A*.
3. (*F*, *A*) is equal to absolute fuzzy soft set, denoted by *U*_*A*_, if *F*(*e*) = *U* for any *e* ∈ *A*.

Clearly, null fuzzy soft set and absolute fuzzy soft set are all classical soft sets. In what follows, we denote by *FS*(*U*, *E*) the set of all fuzzy soft sets over *U* which has a nonempty parameter set.

The concept of similarity measure between two fuzzy sets has been quite studied in the fuzzy literatures. There exist different axiomatic definitions of similarity measures in the literature [30, 33, 34, 48]. These axiomatic definitions depend upon the contexts in which they are constructed. We note that a reasonable similarity measure used for pattern recognition must satisfy at least the following three conditions.

**Definition 5 (see [48]).**

Let *F*(*U*) be the set of all fuzzy subsets on *U*. A function *N* : *F*(*U*) × *F*(*U*)→[0,1] is called a similarity measure if it satisfies the following properties.

(*N*1) *N*(*U*, *∅*) = 0 and *N*(*A*, *A*) = 1 for any *A∈F*(*U*).
(*N*2) *N*(*A*, *B*) = *N*(*B*, *A*) for any *A*, *B∈F*(*U*).
(*N*3) For all *A*, *B*, *C∈F*(*U*), *N*(*A*, *C*) ≤ min*⁡*(*N*(*A*, *B*), *N*(*B*, *C*)) whenever *A⊆B⊆C*.

Li et al. [32] proposed two formulae to calculate the similarity degree between fuzzy sets.

**Theorem 6 (see [32]).**

Given a discrete universe *U* = {*x*_1_,…, *x*_*n*_}, suppose *N*_*θ*_ and *N*_*δ*_ are functions defined for all *A*, *B* ∈ *F*(*U*) by

(2) Nθ(A,B)=(∑i=1n(a−a|A(xi)−B(xi)|+b·min⁡(A(xi),B(xi))))×(∑i=1n(a−(a−1)|A(xi)−B(xi)|+ b·min⁡(A(xi),B(xi))))−1,Nδ(A,B)=(∑i=1n(a−a|A(xi)−B(xi)|+b·min⁡(1−A(xi),1−B(xi))))×(∑i=1n(a−(a−1)|A(xi)−B(xi)| + b·min⁡(1−A(xi),1−B(xi))))−1

with *a* ≥ 0, *b* ≥ 0; then *N*_*θ*_ and *N*_*δ*_ are similarity measures.

Here, in order to avoid the denominator being zero, we set 0/0 = 1. By setting particular values of *a* and *b*, we can obtain some typical similarity measures for fuzzy sets [32].

The similarity measures can be constructed by using fuzzy equivalences and aggregation operators. Fodor and Roubens define fuzzy equivalence as a binary operation on the unit interval in the following way [49].

**Definition 7 (see [49]).**

A function *E* : [0,1]^2^ → [0,1] is called a fuzzy equivalence if it satisfies the following properties.

(*E*1) *E*(*x*, *y*) = *E*(*y*, *x*) for all *x*, *y∈* [0,1].
(*E*2) *E*(*x*, *x*) = 1 for all *x∈* [0,1].
(*E*3) *E*(0,1) = *E*(1,0) = 0.
(*E*4) For all *x*, *y*, *x*′, *y*′ *∈* [0,1], if *x* ≤ *x*′ ≤ *y*′ ≤ *y*, then *E*(*x*, *y*) ≤ *E*(*x*′, *y*′).

It is trivial to prove that (*E*4) is equivalent to the following: for all *x*, *y*, *z* ∈ [0,1], if *x* ≤ *y* ≤ *z*, then *E*(*x*, *z*) ≤ min⁡(*E*(*x*, *y*), *E*(*y*, *z*)).

**Definition 8 (see [50]).**

A function *M* : [0,1]^*n*^ → [0,1] is an aggregation operator if it satisfies the following properties.

(*M*1) *E*(*x*,…, *x*) = *x*.
(*M*2) *M* is monotonic increasing in all of its arguments.

As examples of aggregation operators, we can take the following.

1. The arithmetic mean: *M*(*x*_1_,…, *x*_*n*_) = (1/*n*)∑_*i*=1_^*n*^ *x*_*i*_.
2. The convex linear combinations: *M*(*x*_1_,…, *x*_*n*_) = *λ* · min⁡(*x*_1_,…, *x*_*n*_)+(1 − *λ*)max⁡(*x*_1_,…, *x*_*n*_).
3. *M*(*x*_1_,…, *x*_*n*_) = max⁡(*x*_1_,…, *x*_*n*_)/(max⁡(*x*_1_,…, *x*_*n*_) + max⁡(1 − *x*_1_,…, 1 − *x*_*n*_)).

**Theorem 9 (see [32]).**

Given a discrete universe *U* = {*x*_1_,…, *x*_*n*_}, let *M* be an aggregation operator, *E* a fuzzy equivalence. Suppose *N*_*τ*_ : *F*(*U*) × *F*(*U*)→[0,1] is a function defined for all *A*, *B* ∈ *F*(*U*) by

(3) $$\begin{array}{c} N_{\tau }\left(A,B\right)=M\left(E\left(A\left(x_{1}\right),B\left(x_{1}\right)\right),\ldots ,E\left(A\left(x_{n}\right),B\left(x_{n}\right)\right)\right); \end{array}$$

then *N*_*τ*_ is a similarity measure.

## 3. Similarity Measures for Fuzzy Soft Sets

Similarity measures quantify the extent to which different patterns, images, or sets are alike. Such measures are used extensively in the application of fuzzy sets. Also, it is essential to the application of fuzzy soft set theory. Based on the axioms [48] for a mapping *S* : *F*(*U*) × *F*(*U*)→[0,1] to be a similarity measure between fuzzy sets, we give a definition of a similarity measure for fuzzy soft sets as follows.

**Definition 10 .**

A real function *S* : *FS*(*U*, *E*) × *FS*(*U*, *E*)→[0,1] is called a similarity measure for fuzzy soft sets if it satisfies the following properties.

(*S*1) *S*(*U*_*A*_, *∅*_*A*_) = 0 for any *A⊆E*, and *S*((*F*, *A*), (*F*, *A*)) = 1 for any (*F*, *A*) *∈FS*(*U*, *E*).
(*S*2) *S*((*F*, *A*), (*G*, *B*)) = *S*((*G*, *B*), (*F*, *A*)) for any (*F*, *A*), (*G*, *B*) *∈FS*(*U*, *E*).
(*S*3) For any (*F*, *A*), (*G*, *B*), (*H*, *C*) *∈FS*(*U*, *E*), if (*F*, *A*)*⊆*(*G*, *B*)*⊆*(*H*, *C*), then *S*((*H*, *C*), (*F*, *A*)) ≤ min*⁡*(*S*((*H*, *C*), (*G*, *B*)), *S*((*G*, *B*), (*F*, *A*))).

**Theorem 11 .**

*S*_*θ*_((*F*, *A*), (*G*, *B*)) is a similarity measure, where

(4) $$\begin{array}{c} S_{\theta }\left(\left(F,A\right),\left(G,B\right)\right)=\frac{1}{\left|A\cup B\right|}\underset{e\in A\cap B}{\sum }N_{\theta }\left(F\left(e\right),G\left(e\right)\right) \end{array}$$

for any (*F*, *A*), (*G*, *B*) ∈ *FS*(*U*, *E*).

**Proof**

(*S*1) For any *A*⊆*E* and (*F*, *A*) ∈ *FS*(*U*, *E*), we have

(5) $$\begin{array}{c} S_{\theta }\left(U_{A},\emptyset_{A}\right)=\frac{1}{\left|A\right|}\underset{e\in A}{\sum }N_{\theta }\left(U,\emptyset \right)=0,\\ S_{\theta }\left(\left(F,A\right),\left(F,A\right)\right)=\frac{1}{\left|A\right|}\underset{e\in A}{\sum }N_{\theta }\left(F\left(e\right),F\left(e\right)\right)=\frac{\left|A\right|}{\left|A\right|}=1. \end{array}$$

(*S*2) is trivial.

(*S*3) Let (*F*, *A*), (*G*, *B*), (*H*, *C*) ∈ *FS*(*U*, *E*), and (*F*, *A*)⊆(*G*, *B*)⊆(*H*, *C*). It follows that *A*⊆*B*⊆*C* and *F*(*e*)⊆*G*(*e*)⊆*H*(*e*) for each *e* ∈ *A*. Consequently, *N*_*θ*_(*F*(*e*), *H*(*e*)) ≤ *N*_*θ*_(*F*(*e*), *G*(*e*)), *N*_*θ*_(*F*(*e*), *H*(*e*)) ≤ *N*_*θ*_(*G*(*e*), *H*(*e*)). Thus we have

(6)   Sθ((F,A),(G,B))=1|B|∑e∈ANθ(F(e),G(e))≥1|C|∑e∈ANθ(F(e),H(e))=Sθ((F,A),(H,C)),Sθ((G,B),(H,C))=1|C|∑e∈BNθ(G(e),H(e))≥1|C|∑e∈ANθ(G(e),H(e))≥1|C|∑e∈ANθ(F(e),H(e))=Sθ((F,A),(H,C)).

This completes the proof.

In what follows, we suppose that *U* = {*x*_1_,…, *x*_*n*_}. In (4), let *a* = 0.5,  *b* = 0; then we have

(7) S1((F,A),(G,B)) =1|A∪B|∑e∈A∩B∑i=1n(1−|F(e)(xi)−G(e)(xi)|)∑i=1n(1+|F(e)(xi)−G(e)(xi)|).

Let *a* = 1, *b* = 0; then we have

(8) S2((F,A),(G,B))   =1|A∪B|∑e∈A∩B(1−1n∑i=1n|F(e)(xi)−G(e)(xi)|).

Let *a* = 0, *b* = 1; then we have

(9)   S3((F,A),(G,B)) =1|A∪B|∑e∈A∩B∑i=1nmin⁡(F(e)(xi),G(e)(xi))∑i=1nmax⁡(F(e)(xi),G(e)(xi)).

Let *a* = 0, *b* = 2; then we have

(10) S4((F,A),(G,B)) =1|A∪B|∑e∈A∩B2∑i=1nmin⁡(F(e)(xi),G(e)(xi))∑i=1n(F(e)(xi)+G(e)(xi)).

Wang and Qu [45] proposed a similarity measure for vague soft sets as follows:

(11) $$\begin{array}{c} M\left(\left(F,E\right),\left(G,E\right)\right)=\frac{\sum_{i=1}^{m}M_{i}\left(\left(F,E\right),\left(G,E\right)\right)}{m}, \end{array}$$

where *U* = {*x*_1_,…, *x*_*n*_} is the universal set of elements, *E* = {*e*_1_,…, *e*_*m*_} is the universal set of parameters, *M*_*i*_((*F*, *E*), (*G*, *E*)) = 1 − (1/4*n*)∑_*j*=1_^*n*^(|*S*_*F*(*e*_*i*_)_(*x*_*j*_) − *S*_*G*(*e*_*i*_)_(*x*_*j*_)| + |*t*_*F*(*e*_*i*_)_(*x*_*j*_) − *t*_*G*(*e*_*i*_)_(*x*_*j*_)| + |*f*_*F*(*e*_*i*_)_(*x*_*j*_) − *f*_*G*(*e*_*i*_)_(*x*_*j*_)|), *S*_*F*(*e*_*i*_)_(*x*_*j*_) = *t*_*F*(*e*_*i*_)_(*x*_*j*_) − *f*_*F*(*e*_*i*_)_(*x*_*j*_), and *S*_*G*(*e*_*i*_)_(*x*_*j*_) = *t*_*G*(*e*_*i*_)_(*x*_*j*_) − *f*_*G*(*e*_*i*_)_(*x*_*j*_). In this similarity measure, if (*F*, *E*) and (*G*, *E*) are fuzzy soft sets, that is, *t*_*F*(*e*_*i*_)_(*x*_*j*_) = 1 − *f*_*F*(*e*_*i*_)_(*x*_*j*_), *t*_*G*(*e*_*i*_)_(*x*_*j*_) = 1 − *f*_*G*(*e*_*i*_)_(*x*_*j*_), then we have

(12) M((F,E),(G,E)) =1m∑i=1m(1−1n∑j=1n|tF(ei)(xj)−tG(ei)(xj)|).

We note that this similarity measure is a special case of *S*_2_((*F*, *A*), (*G*, *B*)) if *A* = *B* = *E*.

Majumdar and Samanta [42] proposed a similarity measure based on set theoretic approach as follows. Here some modifications on notations and technical terms have been made to fit the context of our discussion. Let (*F*, *E*) and (*G*, *E*) be two fuzzy soft sets. The similarity measure *M*((*F*, *E*), (*G*, *E*)) is defined as

(13) M((F,E),(G,E)) =max⁡e∈E⁡∑i=1nmin⁡(F(e)(xi),G(e)(xi))∑i=1nmax⁡(F(e)(xi),G(e)(xi)).

This measure does not conform to the idea of similarity measure. For example, let *E* = {*e*_1_, *e*_2_}, *F*(*e*_1_) = *U*, *G*(*e*_1_) = *∅*, *F*(*e*_2_) = *G*(*e*_2_). By

(14) $$\begin{array}{c} \frac{\sum_{i=1}^{n}\min\left(F\left(e_{2}\right)\left(x_{i}\right),G\left(e_{2}\right)\left(x_{i}\right)\right)}{\sum_{i=1}^{n}\max\left(F\left(e_{2}\right)\left(x_{i}\right),G\left(e_{2}\right)\left(x_{i}\right)\right)}=1 \end{array}$$

we have *M*((*F*, *E*), (*G*, *E*)) = 1. On the other hand, *F*(*e*_1_) and *G*(*e*_1_) are quite different. Clearly, *S*_3_ is an improved version of this measure.

**Theorem 12 .**

*S*_*δ*_((*F*, *A*), (*G*, *B*)) is a similarity measure, where

(15) $$\begin{array}{c} S_{\delta }\left(\left(F,A\right),\left(G,B\right)\right)=\frac{1}{\left|A\cup B\right|}\overset{}{\underset{e\in A\cap B}{\sum }}N_{\delta }\left(F\left(e\right),G\left(e\right)\right) \end{array}$$

for any (*F*, *A*), (*G*, *B*) ∈ *FS*(*U*, *E*).

**Proof**

It can be proved in the same manner with Theorem 11.

In (15), let *a* = 0, *b* = 1; then we have

(16) S5((F,A),(G,B)) =1|A∪B|∑e∈A∩B∑i=1nmin⁡(1−F(e)(xi),1−G(e)(xi))∑i=1nmax⁡(1−F(e)(xi),1−G(e)(xi)).

Let *a* = 0, *b* = 2; then we have

(17) S6((F,A),(G,B)) =1|A∪B|∑e∈A∩B2∑i=1nmin⁡(1−F(e)(xi),1−G(e)(xi))∑i=1n(2−F(e)(xi)−G(e)(xi)).

**Theorem 13 .**

*S*_*τ*_((*F*, *A*), (*G*, *B*)) is a similarity measure, where

(18) $$\begin{array}{c} S_{\tau }\left(\left(F,A\right),\left(G,B\right)\right)=\frac{1}{\left|A\cup B\right|}\overset{}{\underset{e\in A\cap B}{\sum }}N_{\tau }\left(F\left(e\right),G\left(e\right)\right) \end{array}$$

for any (*F*, *A*), (*G*, *B*) ∈ *FS*(*U*, *E*).

**Proof**

The proof is similar to that of Theorem 11.

The similarity measure *S*_*τ*_ is determined by a fuzzy equivalence and an aggregation operator. Fuzzy equivalences can be constructed from biresiduations, automorphisms, *t*-norms, and *t*-conorms [49].

An associative, commutative, and increasing function *T* : [0,1]^2^ → [0,1] is called a *t*-norm [51] if it has the neutral element equal to 1; that is, *T*(1, *x*) = *x* for each *x* ∈ [0,1]. The residuation of a *t*-norm *T* is the function $\vec{T}:{\left[0,1\right]}^{2}\to [0,1]$ defined for all *x*, *y* ∈ [0,1] by $\vec{T}(x,y)=\sup\{\alpha \in [0,1];\;T(x,\alpha )\leq y\}$. It has been proved that the biresiduation *E*_*T*_ of a *t*-norm *T* is a fuzzy equivalence [49], where $E_{T}(x,y)=\min(\vec{T}(x,y),\vec{T}(y,x))$.

Three typical *t*-norms are *T*_*M*_(*x*, *y*) = min⁡(*x*, *y*), *T*_*P*_(*x*, *y*) = *xy*, and *T*_*L*_(*x*, *y*) = max⁡(*x* + *y* − 1,0). The corresponding fuzzy equivalences are as follows.

1. *E*_*T*_*M*__(*x*, *y*) = min⁡(*x*, *y*) whenever *x* ≠ *y*, and *E*_*T*_*M*__(*x*, *y*) = 1 otherwise.
2. *E*_*T*_*P*__(*x*, *y*) = min⁡(*x*, *y*)/max⁡(*x*, *y*).
3. *E*_*T*_*L*__(*x*, *y*) = 1 − |*x* − *y*|.

A continuous, strictly increasing function *φ* : [0,1]^2^ → [0,1] with boundary conditions *φ*(0) = 0 and *φ*(1) = 1 is called an automorphism [52] of the interval [0,1]. Let *E* be a fuzzy equivalence, *φ* an automorphism of [0,1]. Then the function *E*_*φ*_ : [0,1]^2^ → [0,1] is a fuzzy equivalence [32], where *E*_*φ*_(*x*, *y*) = *E*(*φ*(*x*), *φ*(*y*)) for all *x*, *y* ∈ [0,1]. Taking the automorphisms *φ*_1_(*x*) = 2*x*/(1 + *x*),  *φ*_2_(*x*) = *x* ^2^ and the fuzzy equivalence *E*_*T*_*L*__, we obtain the following fuzzy equivalences.

(4) *E*_1_(*x*, *y*) = 1 − |2*x*/(1 + *x*) − 2*y*/(1 + *y*)| = 1 − 2|*x* − *y*|/((1 + *x*)(1 + *y*)).
(5) *E*_2_(*x*, *y*) = 1 − |*x* ^2^ − *y* ^2^|.

If we take the arithmetic mean aggregation operator *M* and the fuzzy equivalences *E*_*T*_*M*__, *E*_*T*_*P*__, *E*_*T*_*L*__, *E*_1_, and *E*_2_, respectively, then we have the following similarity measures for fuzzy soft sets:

(19) S7((F,A),(G,B)) =1n|A∪B|∑e∈A∩B∑  i=1nETM(F(e)(xi),G(e)(xi)),S8((F,A),(G,B)) =1n|A∪B|∑e∈A∩B∑  i=1nmin⁡⁡(F(e)(xi),G(e)(xi))max⁡⁡(F(e)(xi),G(e)(xi)),S9((F,A),(G,B)) =1n|A∪B|∑e∈A∩B∑  i=1n(1−|F(e)(xi)−G(e)(xi)|),S10((F,A),(G,B)) =1|A∪B|  ×∑e∈A∩B(1−1n∑i=1n2|F(e)(xi)−G(e)(xi)|(1+F(e)(xi))(1+G(e)(xi))),S11((F,A),(G,B)) =1n|A∪B|∑e∈A∩B∑  i=1n(1−|(F(e)(xi))2−(G(e)(xi))2|).

We note that *S*_9_ = *S*_2_.

In the constructions of the similarity measures *S*_*θ*_, *S*_*δ*_, and *S*_*τ*_, for fuzzy soft sets (*F*, *A*), (*G*, *B*), we only compare *F*(*e*) and *G*(*e*) for common parameter *e* ∈ *A*∩*B*. The parameters *e* ∈ (*A* − *B*)∪(*B* − *A*) have not been taken into account. According to the idea of soft equality [8], we may think that *G*(*e*) = *∅* whenever *e* ∈ *A* − *B* and *F*(*e*) = *∅* whenever *e* ∈ *B* − *A*. Based on this observation, we propose the following similarity measures.

**Theorem 14 .**

*S*_*θ*_′((*F*, *A*), (*G*, *B*)) is a similarity measure, where

(20) Sθ′((F,A),(G,B)) =1|A∪B|  ×(∑e∈A∩BNθ(F(e),G(e))  +min⁡{∑e∈A−BNθ(F(e),∅),∑e∈B−ANθ(∅,G(e))})

for any (*F*, *A*), (*G*, *B*) ∈ *FS*(*U*, *E*).

We note that *S*_*θ*_′((*F*, *A*), (*G*, *B*)) = *S*_*θ*_((*F*, *A*), (*G*, *B*)) if *A*⊆*B*. Thus the proof of this theorem is similar to that of Theorem 11.

Similar to Theorem 14 we obtain the following two theorems.

**Theorem 15 .**

*S*_*δ*_′((*F*, *A*), (*G*, *B*)) is a similarity measure, where

(21) Sδ′((F,A),(G,B))=1|A∪B| ×(∑e∈A∩BNδ(F(e),G(e))  +min⁡{∑e∈A−BNδ(F(e),∅),∑e∈B−ANδ(∅,G(e))})

for any (*F*, *A*), (*G*, *B*) ∈ *FS*(*U*, *E*).

**Theorem 16 .**

*S*_*τ*_′((*F*, *A*), (*G*, *B*)) is a similarity measure, where

(22) Sτ′((F,A),(G,B))=1|A∪B| ×(∑e∈A∩BNτ(F(e),G(e))  +min⁡⁡{∑e∈A−BNτ(F(e),∅),∑e∈B−ANτ(∅,G(e))})

for any (*F*, *A*), (*G*, *B*) ∈ *FS*(*U*, *E*).

From Theorems 14, 15, and 16, we can obtain similarity measure *S*_*i*_′ in the same manner with *S*_*i*_  (1 ≤ *i* ≤ 11). For example,

(23) S1′((F,A),(G,B)) =1|A∪B|(∑e∈A∩B∑i=1n(1−|F(e)(xi)−G(e)(xi)|)∑i=1n(1+|F(e)(xi)−G(e)(xi)|)+min⁡{∑e∈A−B∑i=1n(1−F(e)(xi))∑i=1n(1+F(e)(xi)),∑e∈B−A∑i=1n(1−G(e)(xi))∑i=1n(1+G(e)(xi))}),S2′((F,A),(G,B)) =1|A∪B|(∑e∈A∩B(1−1n ×∑i=1n|F(e)(xi)−G(e)(xi)|)+min⁡{∑e∈A−B(1−1n∑i=1nF(e)(xi)),∑e∈B−A(1−1n∑i=1nG(e)(xi))  }),S3′((F,A),(G,B)) =1|A∪B|∑e∈A∩B∑i=1nmin⁡(F(e)(xi),G(e)(xi))∑i=1nmax⁡(F(e)(xi),G(e)(xi)),S4′((F,A),(G,B)) =1|A∪B|∑e∈A∩B2∑i=1nmin⁡(F(e)(xi),G(e)(xi))∑i=1n(F(e)(xi)+G(e)(xi)).

We note that *S*_3_′((*F*, *A*), (*G*, *B*)) = *S*_3_((*F*, *A*), (*G*, *B*)), *S*_4_′((*F*, *A*), (*G*, *B*)) = *S*_4_((*F*, *A*), (*G*, *B*)).

**Example 17 .**

Let *U* = {*x*_1_, *x*_2_, *x*_3_, *x*_4_}, *E* = {*e*_1_, *e*_2_, *e*_3_, *e*_4_}, *A* = {*e*_1_, *e*_3_, *e*_4_}, and *B* = {*e*_2_, *e*_3_, *e*_4_}. Suppose that (*F*, *A*) and (*G*, *B*) are fuzzy soft sets over *U* given by

(24) $$\begin{array}{c} F\left(e_{1}\right)=\frac{0.2}{x_{1}}+\frac{0.1}{x_{2}}+\frac{0.5}{x_{3}}+\frac{0.1}{x_{4}},\\ F\left(e_{3}\right)=\frac{0.9}{x_{1}}+\frac{0.6}{x_{2}}+\frac{0.3}{x_{3}}+\frac{0.3}{x_{4}},\\ F\left(e_{4}\right)=\frac{1}{x_{1}}+\frac{0.5}{x_{2}}+\frac{0.2}{x_{3}}+\frac{0.4}{x_{4}},\\ G\left(e_{2}\right)=\frac{0.3}{x_{1}}+\frac{0.5}{x_{2}}+\frac{0.3}{x_{3}}+\frac{0.9}{x_{4}},\\ G\left(e_{3}\right)=\frac{0.2}{x_{1}}+\frac{0.2}{x_{2}}+\frac{0.2}{x_{3}}+\frac{0.8}{x_{4}},\\ G\left(e_{4}\right)=\frac{0.9}{x_{1}}+\frac{0.1}{x_{2}}+\frac{0.1}{x_{3}}+\frac{0.7}{x_{4}}. \end{array}$$

By the definition, we have

(25) S1((F,A),(G,B)) =1|A∪B|∑e∈A∩B∑i=1n(1−|F(e)(xi)−G(e)(xi)|)∑i=1n(1+|F(e)(xi)−G(e)(xi)|) =14(∑i=1n(1−|F(e3)(xi)−G(e3)(xi)|)∑i=1n(1+|F(e3)(xi)−G(e3)(xi)|)+∑i=1n(1−|F(e4)(xi)−G(e4)(xi)|)∑i=1n(1+|F(e4)(xi)−G(e4)(xi)|)) =14(0.3+0.6+0.9+0.51.7+1.4+1.1+1.5+0.9+0.6+0.9+0.71.1+1.4+1.1+1.3)≈0.26.

Similarly,

(26) $$\begin{array}{c} S_{2}\left(\left(F,A\right),\left(G,B\right)\right)\approx 0.34,\\ S_{1}^{\prime }\left(\left(F,A\right),\left(G,B\right)\right)\approx 0.34,\\ S_{2}^{\prime }\left(\left(F,A\right),\left(G,B\right)\right)\approx 0.46. \end{array}$$

## 4. Entropy for Fuzzy Soft Sets

The entropy quantifies the degree of uncertainty. In 1965, Zadeh introduced the fuzzy entropy for the first time [39]. de Luca and Termini [34] introduced the axiom construction of entropy of fuzzy sets. There are several entropies of fuzzy sets proposed in the literature [30, 33, 34, 37, 38, 40]. Based on the axioms for the entropy of fuzzy sets [34], we give the definition of entropy for fuzzy soft sets as follows.

**Definition 18 .**

A real function *E* : *FS*(*U*, *E*)→[0, +*∞*) is called an entropy on *FS*(*U*, *E*), if *E* has the following properties.

(*E*1) *E*(*F*, *A*) = 0 if (*F*, *A*) is a soft set.
(*E*2) *E*(*F*, *A*) = 1 if *F*(*e*) = [0.5] for any *e∈A*, where [0.5] is the fuzzy set with the membership function [0.5](*x*) = 0.5 for each *x∈* *U*.
(*E*3) Let (*F*, *A*) be crisper than (*G*, *A*); that is, for any *e∈A* and *x∈* *U*, *F*(*e*)(*x*) ≤ *G*(*e*)(*x*) if *G*(*e*)(*x*) ≤ 0.5 and *F*(*e*)(*x*) ≥ *G*(*e*)(*x*) if *G*(*e*)(*x*) ≥ 0.5. Then *E*(*F*, *A*) ≤ *E*(*G*, *A*).
(*E*4) *E*(*F*, *A*) = *E*(*F* ^*c*^, *A*), where (*F* ^*c*^, *A*) is the complement of fuzzy soft set (*F*, *A*) given by *F* ^*c*^(*e*) = (*F*(*e*))^*c*^ for each *e∈A*.

**Theorem 19 .**

*E*_*θ*_(*F*, *A*) is an entropy, where

(27) $$\begin{array}{c} E_{\theta }\left(F,A\right)=S_{\theta }\left(\left(F,A\right),\left(F^{c},A\right)\right) \end{array}$$

for any (*F*, *A*) ∈ *FS*(*U*, *E*).

**Proof**

(*E*1) We assume that (*F*, *A*) is a soft set. It follows that, for each *e* ∈ *A* and *x*_*i*_ ∈ *U*, *F*(*e*)(*x*_*i*_) = 0 or *F*(*e*)(*x*_*i*_) = 1. Consequently,

(28) a−a|2F(e)(xi)−1|  +b·min⁡(F(e)(xi),1−F(e)(xi))=0,

(29) Nθ(F(e),Fc(e)) =(∑i=1n(a−a|2F(e)(xi)−1|+b·min⁡(F(e)(xi),1−F(e)(xi))))  ×(∑i=1n(a−(a−1)|2F(e)(xi)−1|+b·min⁡(F(e)(xi),1−F(e)(xi))))−1 =0.

Thus we have

(30) Eθ(F,A)=Sθ((F,A),(Fc,A))=1|A|∑e∈ANθ(F(e),Fc(e))=0.

(*E*2) We assume that *F*(*e*) = [0.5] for any *e* ∈ *A*. It follows that *F*(*e*) = *F* ^*c*^(*e*) and hence *N*_*θ*_(*F*(*e*), *F* ^*c*^(*e*)) = 1. Thus we have *E*_*θ*_(*F*, *A*) = *S*_*θ*_((*F*, *A*), (*F* ^*c*^, *A*)) = 1.

(*E*3) Let (*F*, *A*) be crisper than (*G*, *A*). By the definition, we have

(31) Nθ(F(e),Fc(e)) =(∑i=1n(a−a|2F(e)(xi)−1|+b·min⁡(F(e)(xi),1−F(e)(xi))))  ×(∑i=1n(a−(a−1)|2F(e)(xi)−1|+b·min⁡(F(e)(xi),1−F(e)(xi))))−1,Nθ(G(e),Gc(e)) =(∑i=1n(a−a|2G(e)(xi)−1|+b·min⁡(G(e)(xi),1−G(e)(xi))))  ×(∑i=1n(a−(a−1)|2G(e)(xi)−1|  + b·min⁡(G(e)(xi),1−G(e)(xi))))−1.

Consider the following function:

(32) f(y1,…,yn) =∑i=1n(a−a|2yi−1|+b·min⁡(yi,1−yi))∑i=1n(a−(a−1)|2yi−1|+b·min⁡(yi,1−yi)),

where *y*_*i*_ ∈ [0,1]. If *y*_*j*_ ∈ [0,0.5], then we have

(33) ∂f∂yj=b∑i=1n|2yi−1|+2∑i=1n(a+bmin⁡(yi,1−yi))[∑i=1n(a−(a−1)|2yi−1|+bmin⁡(yi,1−yi))]2≥0.

If *y*_*j*_ ∈ [0.5,1], then we have

(34) ∂f∂yj=−b∑i=1n|2yi−1|−2∑i=1n(a+bmin⁡(yi,1−yi))[∑i=1n(a−(a−1)|2yi−1|+bmin⁡(yi,1−yi))]2≤0.

Therefore, we can conclude that *f* is increasing with respect to *y*_*j*_  (*j* = 1,…, *n*) if *y*_*j*_ ∈ [0,0.5], and *f* is decreasing with respect to *y*_*j*_  (*j* = 1,…, *n*) if *y*_*j*_ ∈ [0.5,1]. We note that *F*(*e*)(*x*_*i*_) ≤ *G*(*e*)(*x*_*i*_) if *G*(*e*)(*x*_*i*_) ≤ 0.5 and *F*(*e*)(*x*_*i*_) ≥ *G*(*e*)(*x*_*i*_) if *G*(*e*)(*x*_*i*_) ≥ 0.5. Thus we have *N*_*θ*_(*F*(*e*), *F* ^*c*^(*e*)) ≤ *N*_*θ*_(*G*(*e*), *G* ^*c*^(*e*)) and, consequently,

(35) Eθ(F,A)=Sθ((F,A),(Fc,A))=1|A|∑e∈ANθ(F(e),Fc(e))≤1|A|∑e∈ANθ(G(e),Gc(e))=Sθ((G,A),(Gc,A))=Eθ(G,A).

(*E*4) is trivial.

By the definition, we have *N*_*δ*_(*A*, *B*) = *N*_*θ*_(*A* ^*c*^, *B* ^*c*^). It follows that *N*_*δ*_(*A*, *A* ^*c*^) = *N*_*θ*_(*A*, *A* ^*c*^) and hence *S*_*δ*_((*F*, *A*), (*F* ^*c*^, *A*)) = *S*_*θ*_((*F*, *A*), (*F* ^*c*^, *A*)) for any (*F*, *A*) ∈ *FS*(*U*, *E*). Thus we have the following corollary.

**Corollary 20 .**

*E*_*δ*_(*F*, *A*) is an entropy, where

(36) $$\begin{array}{c} E_{\delta }\left(F,A\right)=S_{\delta }\left(\left(F,A\right),\left(F^{c},A\right)\right) \end{array}$$

for any (*F*, *A*) ∈ *FS*(*U*, *E*).

**Theorem 21 .**

*E*_*τ*_(*F*, *A*) is an entropy, where

(37) $$\begin{array}{c} E_{\tau }\left(F,A\right)=S_{\tau }\left(\left(F,A\right),\left(F^{c},A\right)\right) \end{array}$$

for any (*F*, *A*) ∈ *FS*(*U*, *E*).

**Proof**

(*E*1) We assume that (*F*, *A*) is a soft set. It follows that, for each *e* ∈ *A* and *x*_*i*_ ∈ *U*, *F*(*e*)(*x*_*i*_) = 0 or *F*(*e*)(*x*_*i*_) = 1. Consequently,

(38) $$\begin{array}{c} E\left(F\left(e\right)\left(x_{i}\right),1-F\left(e\right)\left(x_{i}\right)\right)=E\left(0,1\right)=0,\\ N_{\tau }\left(F\left(e\right),F^{c}\left(e\right)\right)=M\left(0,\ldots ,0\right)=0. \end{array}$$

Thus we have *E*_*τ*_(*F*, *A*) = 0.

(*E*2) We assume that *F*(*e*) = [0.5] for any *e* ∈ *A*. It follows that *F*(*e*) = *F* ^*c*^(*e*) and hence *F*(*e*)(*x*_*i*_) = *F* ^*c*^(*e*)(*x*_*i*_) = 0.5. Thus we have

(39) $$\begin{array}{c} E\left(F\left(e\right)\left(x_{i}\right),F^{c}\left(e\right)\left(x_{i}\right)\right)=E\left(0.5,0.5\right)=1,\\ N_{\tau }\left(F\left(e\right),F^{c}\left(e\right)\right)=M\left(1,\ldots ,1\right)=1. \end{array}$$

Consequently, *E*_*τ*_(*F*, *A*) = *S*_*τ*_((*F*, *A*), (*F* ^*c*^, *A*)) = 1.

(*E*3) Let (*F*, *A*) be crisper than (*G*, *A*). For any *e* ∈ *A*, we have

(40) Nτ(F(e),Fc(e)) =M(E(F(e)(x1),1−F(e)(x1)),…, E(F(e)(xn),1−F(e)(xn))),Nτ(G(e),Gc(e)) =M(E(G(e)(x1),1−G(e)(x1)),…, E(G(e)(xn),1−G(e)(xn))).

If *G*(*e*)(*x*_*i*_) ≤ 0.5, then *F*(*e*)(*x*_*i*_) ≤ *G*(*e*)(*x*_*i*_) and hence

(41) $$\begin{array}{c} F\left(e\right)\left(x_{i}\right)\leq G\left(e\right)\left(x_{i}\right)\leq 1-G\left(e\right)\left(x_{i}\right)\leq 1-F\left(e\right)\left(x_{i}\right). \end{array}$$

If *G*(*e*)(*x*_*i*_) ≥ 0.5, then *F*(*e*)(*x*_*i*_) ≥ *G*(*e*)(*x*_*i*_) and hence

(42) $$\begin{array}{c} 1-F\left(e\right)\left(x_{i}\right)\leq 1-G\left(e\right)\left(x_{i}\right)\leq G\left(e\right)\left(x_{i}\right)\leq F\left(e\right)\left(x_{i}\right). \end{array}$$

It follows that *E*(*F*(*e*)(*x*_1_), 1 − *F*(*e*)(*x*_1_)) ≤ *E*(*G*(*e*)(*x*_1_), 1 − *G*(*e*)(*x*_1_)). We note that *M* is increasing in all of its arguments. Thus *N*_*τ*_(*F*(*e*), *F* ^*c*^(*e*)) ≤ *N*_*τ*_(*G*(*e*), *G* ^*c*^(*e*)) and in consequence *E*_*τ*_(*F*, *A*) ≤ *E*_*τ*_(*G*, *A*).

(*E*4) is trivial.

Using similarity measures *S*_*i*_  (1 ≤ *i* ≤ 11), we can obtain the corresponding entropies *E*_*i*_  (1 ≤ *i* ≤ 11). For example, we have

(43) E1(F,A)=1|A|∑e∈A∑i=1n(1−|2F(e)(xi)−1|)∑i=1n(1+|2F(e)(xi)−1|),E2(F,A)=1|A|∑e∈A(1−1n∑i=1n|2F(e)(xi)−1|),E3(F,A)=1|A|∑e∈A∑i=1nmin⁡(F(e)(xi),1−F(e)(xi))∑i=1nmax⁡(F(e)(xi),1−F(e)(xi)),E4(F,A)=2n|A|∑e∈A∑  i=1nmin⁡(F(e)(xi),1−F(e)(xi)),E8(F,A)=1n|A|∑e∈A∑  i=1nmin⁡(F(e)(xi),1−F(e)(xi))max⁡(F(e)(xi),1−F(e)(xi)),E11(F,A)=1n|A|∑e∈A∑  i=1n(1−|2F(e)(xi)−1|).

We note that *E*_2_ = *E*_11_.

## 5. Concluding Remarks

Soft set theory was originally proposed as a general mathematical tool for dealing with uncertainties. Majumdar and Samanta [41, 42] initiated the study of uncertainty measures of soft sets and fuzzy soft sets. This paper is devoted to a further discussion along this line. The axioms for similarity measure and entropy of fuzzy soft sets are proposed. Based on fuzzy equivalences, a new category of similarity measures and entropies is presented. The relationships among these measures and the other proposals in the literature are analyzed. Based on these uncertainty measures, we can further probe the applications of fuzzy soft sets in the fields such as pattern recognition, data analysis, and decision making. The extension of our approach to intuitionistic fuzzy soft sets and vague soft sets is another important and interesting issue to be addressed.
